# Cyclodextrin-Based Functional Glyconanomaterials

**DOI:** 10.3390/nano10122517

**Published:** 2020-12-15

**Authors:** Gonzalo Rivero-Barbarroja, Juan Manuel Benito, Carmen Ortiz Mellet, José Manuel García Fernández

**Affiliations:** 1 Department of Organic Chemistry, Faculty of Chemistry, University of Seville, 41012 Seville, Spain; grbarbarroja@us.es (G.R.-B.); mellet@us.es (C.O.M.); 2 Instituto de Investigaciones Químicas (IIQ), CSIC, Universidad de Sevilla, 41092 Sevilla, Spain; juanmab@iiq.csic.es

**Keywords:** cyclodextrins, drug delivery, gene delivery, glyconanomaterials, glyconanoparticles, glycopolymers, glycotargeting, multivalency, self-assembly, supramolecular nanomaterials

## Abstract

Cyclodextrins (CDs) have long occupied a prominent position in most pharmaceutical laboratories as “off-the-shelve” tools to manipulate the pharmacokinetics of a broad range of active principles, due to their unique combination of biocompatibility and inclusion abilities. The development of precision chemical methods for their selective functionalization, in combination with “click” multiconjugation procedures, have further leveraged the nanoscaffold nature of these oligosaccharides, creating a direct link between the glyco and the nano worlds. CDs have greatly contributed to understand and exploit the interactions between multivalent glycodisplays and carbohydrate-binding proteins (lectins) and to improve the drug-loading and functional properties of nanomaterials through host–guest strategies. The whole range of capabilities can be enabled through self-assembly, template-assisted assembly or covalent connection of CD/glycan building blocks. This review discusses the advancements made in this field during the last decade and the amazing variety of functional glyconanomaterials empowered by the versatility of the CD component.

## 1. Introduction

Cyclodextrins (CDs) are cyclic oligosaccharides with unique inside/outside distribution of hydrophobic/hydrophilic areas, resulting in well-defined topological amphiphilicity. This property confers CDs distinctive advantages to form inclusion complexes with lipophilic guest molecules as far as them entirely or partially entering the CD macro-ring cavity. The stability and water solubility of the guest can be improved in this manner, or the target guest can be captured, removed or masked, broadening application scopes. Indeed, the academic and (bio)technological interest for cyclodextrins has been historically dominated by their inclusion complex-formation ability, which has translated into numerous applications in fields like food [1,2], cosmetics [3], environment [4,5], and medicine [3,6,7,8,9,10].

**Figure 1:**
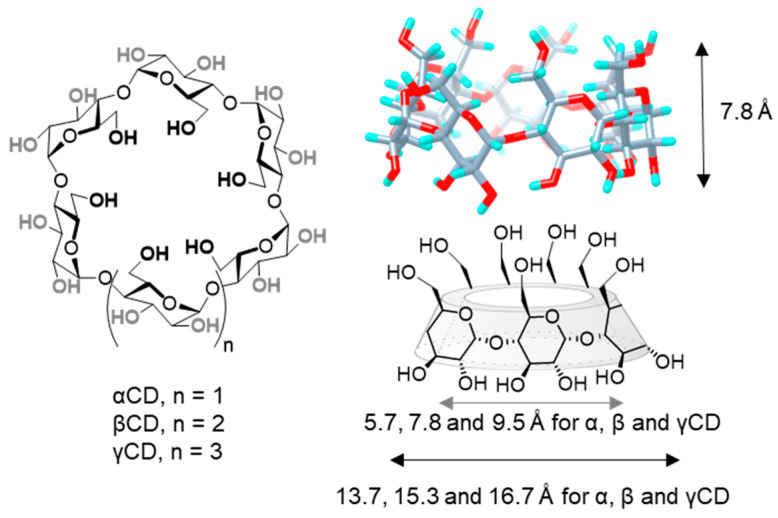
Structures of α, β and γCD (left), 3D view of βCD (upper-right) and schematic representation of CD basket-shape architecture (lower right), with indication of the height, and average internal and external diameters for the three commercially available representatives. CD, cyclodextrin.

Structurally, CDs are composed by α(1→4)-linked glucose units that feature a characteristic toroidal truncated-cone shape, in which glucose hydroxyls orient to the outer space flanking the upper and lower rims, while methinic protons (H-5 and H-3) point to the inner cavity. The hexamer (αCD), heptamer (βCD) and octamer (γCD) representatives are commercially available. All three are nanometric objects that share the same torus height (7.8 Å), as defined by the monosaccharide dimensions, and differ on the external (13.7, 15.3 and 16.7 Å) and internal diameters (5.7, 7.8 and 9.5 Å, respectively), the later determining their affinity towards size-variable guests (Figure 1) [11,12]. Conceptually, CDs can be considered as molecular nanoparticles in the sense advanced by Cheng and coworkers: nanometric molecular systems of natural or technological origin exhibiting persistent topology (shape and volume) [13,14]. They are susceptible to selective chemical manipulation to obtain sugary molecular nanosystems with tailored capabilities, or they can be used as functional components to engineer CD-based nanosystems benefiting from the CD inclusion properties [15,16,17]. Alternatively, they can be incorporated into polymeric, magnetic, lipid, metallic or mesoporous NPs to enhance their characteristics [18,19]. All these examples highlight the potential of CDs to galvanize positive synergies between research disciplines, such as synthetic chemistry, supramolecular chemistry, pharmaceutical formulation or materials science.

Strictly speaking, CD-based nanomaterials can be considered as functional glyconanomaterials (CDs are definitely *glyco*) [20]. However, in the context of this thematic issue, this concept is reserved for nanomaterials exposing carbohydrate moieties mediating interactions with other biomolecular partners. By CD-based functional glyconanomaterials, here, we mean nanosized materials combining CD and glycan modules, either with an organic or inorganic core, obtained by self-assembly, template-assisted assembly or covalent conjugation. All these strategies will be discussed in this review, with an emphasis in the properties and applications of the resulting hybrid nanoconstructs. Note that molecular CD-glycan conjugates, widely used in fundamental studies on carbohydrate–protein interactions and in the design of glycotargeted CD drug carriers, are purposely excluded for the sake of consistency. Readers are eagerly encouraged to consult more comprehensive accounts on these topics for a wider view of the state-of-the-art [21,22,23,24,25]. The fabrication of glycosurfaces by exploiting (glyco)CD chemical and supramolecular properties is also not covered, and readers interested in this particular area of CD-based glycotechnology are addressed to recent reviews [16,26,27] and key contributions in the field [28,29,30,31].

## 2. Multitopic Guest-Driven Clusterization of GlycoCDs

**Figure 2:**
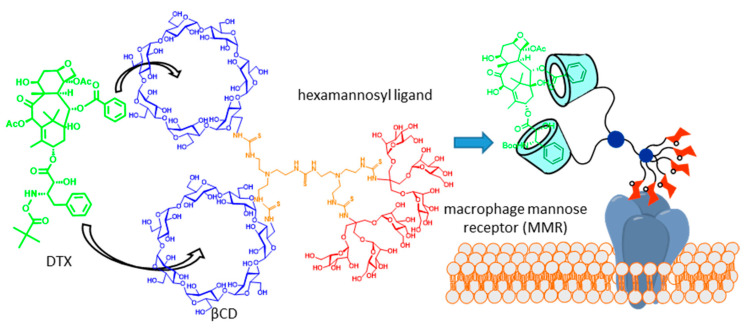
Docetaxel (DTX) carrier consisting of a hexamannosylated dimeric βCD derivative designed on the basis of the drug clusterization concept [35]. Adapted with permission from Reference [3]. Copyright 2013 Royal Society of Chemistry.

Molecular scaffolds displaying two or more CD cavity-fitting motifs can behave, in principle, as multitopic guests, with the potential of forming high-order inclusion complexes. If the CD partner is a glycoCD, the total glycoligand valency of the resulting supramolecular species will grow with complex stoichiometry, which is expected to enhance the binding affinity towards complementary lectin receptors by virtue of the so-called multivalent or cluster effect [32,33,34]. The potential of this strategy in glycotargeting schemes was anticipated in 2004, in the context of a work aiming at developing an efficient carrier for the delivery of the taxane anticancer drug docetaxel (DTX, Taxotère^®^) to macrophages [35]. DTX possesses two phenyl fragments that are well-suited for βCD inclusion, whereas active uptake by macrophages can be promoted by targeting the macrophage mannose receptor (MMR). On such grounds, a trivalent βCD-mannosyl dendron conjugate was initially conceived. MMR binding studies evidenced a statistically significant increase in affinity in the presence of the drug, which is in agreement with the DTX-promoted clusterization of the conjugate. The association constant (*K*_a_) of phenyl derivatives and βCD (*K*_a_ ≈ 10^3^ M) [36] is, however, too modest to warrant a medically useful concentration of the 2:1 complex in biological media, which was overcome by engineering a dimeric βCD-hexamannosyl dendron conjugate that formed a very strong 1:1 chelate-type complex with DTX (Figure 2).

**Figure 3:**
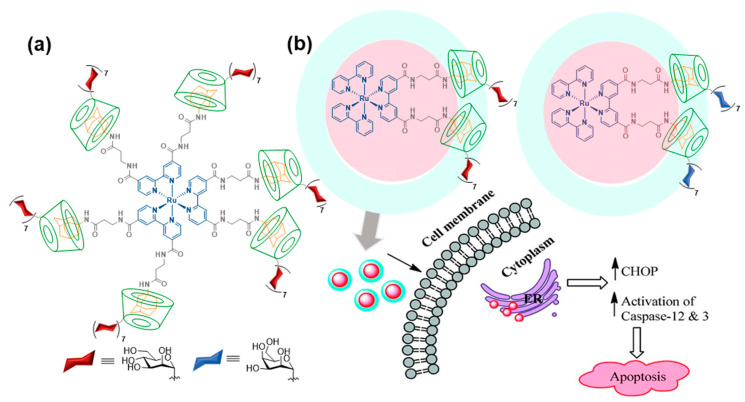
Heptamannosyl-βCD/Ru(II)-scaffolded hexa-adamantyl host–guest complex reported by Seeberger and coworkers [38] (**a**) and bis-adamantyl analogs with anti-apoptotic activity reported by Kikkeri and coworkers [40] (**b**). Adapted with permission from Reference [40]. Copyright 2016 Royal Society of Chemistry.

Adamantane (Ad) derivatives form much more robust inclusion complexes with βCD hosts than phenyl derivatives (*K*_a_ ≈ 10^5^ M). This property has been amply used for macromolecular materials design [37]. Notwithstanding, reports on βCD/Ad host–guest supramolecular ligation chemistry using discrete molecular entities, for the purpose of assembling functional glyconanomaterials, are relatively scarce. In 2011, Seeberger and coworkers showed that the incorporation of up to six Ad moieties onto a fluorescent ruthenium(II)-bipyridine complex core provided a very convenient platform for the rapid construction of high-valent glyconanosensors for the visualization of carbohydrate–lectin interactions, just by mixing with sugar-appended βCD derivatives (Figure 3a) [38]. A follow-up of this proof of concept was reported by Kikkeri and coworkers [39], who synthesized the two enantiomerically pure versions of the hexa-Ad guest (denoted Δ and Λ for the positive and negative sign of the circular dichroism signal, respectively) and used them to build diastereomeric metallo-glycodendrimers by anchoring a heptamannosylated βCD conjugate. Evaluation of the binding affinities towards different mannose-binding C-type lectins, including the human Dendritic Cell-Specific Intercellular adhesion molecule-3-Grabbing Non-integrin (CD-SIGN), the mouse-Specific Intracellular adhesion molecule-3 Grabbing Non-integrin homolog-Related 3 (SIGNR3) and Dectin-1, evidenced amazing differences that revealed a major impact of the chiral microenvironment in the recognition process, in spite of the similar topology of the supramolecular diastereomers. Such dissimilar behaviors were also confirmed in cellulo as well as in vivo. The same authors prepared metallo-glycodendrimers from a Ru(II) bipyridine complex that has attached only two Ad motifs and either a heptamannosylated or a heptagalactosylated βCD component [40]. The glycodendrimers demonstrated selective carbohydrate–protein interaction properties and mediated the delivery of the Ru(II) complexes into cancer cells expressing specific mannose- or galactose-selective lectins, respectively. Once internalized, they showed cytotoxic activity by interacting with the endoplasmic reticulum (ER) and triggering caspase-mediated apoptosis, whereas they were not cytotoxic to normal cells (Figure 3b).

**Figure 4:**
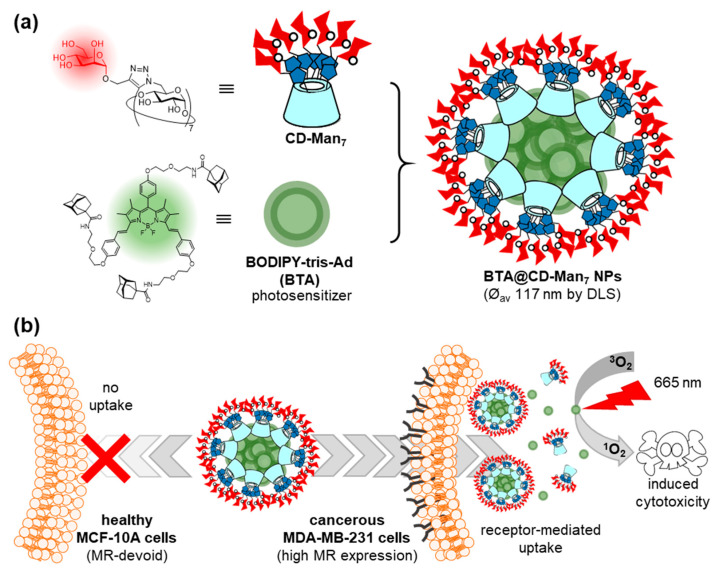
Structures of the CD-Man_7_ glycoCD host and BTA tritopic guest used by Zhang, Yin and coworkers (**a**) and schematic representation of their self-assembly into nanoaggregates for targeted photodynamic therapy against breast cancer (**b**) [42]. CD, cyclodextrin; Man, mannose; BODIPY, boron-dipyrromethene; BTA, BODIPY-tris-Ad derivative; LSD, dynamic light scattering; MR, mannose receptor.

Ferrocene and boron-dipyrromethene (BODIPY) have also been used as core elements for the construction of Ad guests, in order to clusterize glycoCDs into functional glyconanomaterials through inclusion complex formation. For instance, a ferrocene-bis-Ad guest was found to form a supramolecular dimer with a heptalactosyl-βCD derivative that was employed to assess binding to the legume lectin peanut agglutinin (PNA), broadly used in fundamental studies on carbohydrate–protein recognition [41]. Zhang, Yin and coworkers prepared a BODIPY-tris-Ad derivative (BTA) and used it to prepare mannose-coated nanoparticles by the spontaneous supramolecular immobilization of a heptamannosylated-βCD conjugate (CD-Man_7_) onto the surface of BTA aggregates upon co-precipitation (Figure 4) [42]. Transmission electron microscopy (TEM) revealed the formation of spherical BTA@CD-Man_7_ nanoparticles with an average diameter of 117 ± 16 nm, whereas confocal laser scanning microscopy (CLMS) showed that the nanoparticles were efficiently internalized in MDA-MB-231 breast cancer cells overexpressing the mannose receptor (MR), but not in healthy MCF-10A cells. The nanoparticles accumulated in the lysosomes of the cancer cells, where they dissociated and revealed the photosensitizer (PS) character of BODIPY. Irradiation with 665 nm light emitting diode (LED) light then resulted in phototoxicity. The potential of the mannose-mediated PS system for targeted photodynamic therapy (PDT) was confirmed in a mouse model that was established by injecting MDA-MB-231 cancer cells into subcutaneous tissues, confirming the remarkable tumor inhibition effect of BTA@CD-Man_7_ under irradiation.

## 3. Amphiphilicity as an Action Principle for GlycoCD Self-Assembly

**Figure 5:**
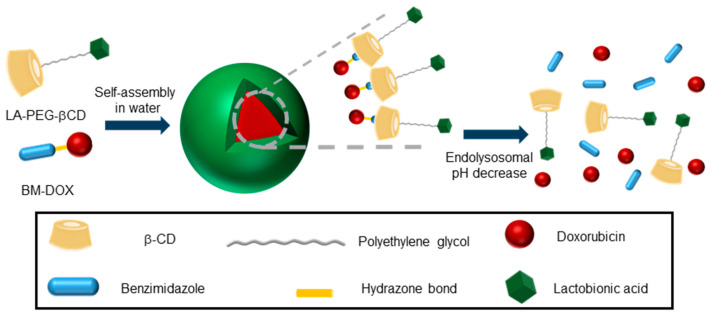
Schematic illustration of the superamphiphile strategy developed by Zhang and coworkers for the self-assembly of acid-sensitive ASGPR-targeted prodrug glyconanoparticles [46]. ASGPR, asialoglycoprotein receptor; LA, lactobionic acid; PEG, poly(ethylenglycol); βCD, β-cyclodextrin; BM, benzimidazole; DOX, doxorubicin.

Inclusion complex formation can be exploited to impart self-assembling properties to CD derivatives beyond multitopic guest-driven clusterization. For instance, if the guested molecule contains a hydrophobic tail, the resulting species will behave as a supramolecular amphiphile or “superamphiphile”, a concept that emerged during the last decade and refers to surfactants where the hydrophilic and hydrophobic moieties are linked by noncovalent connections [43]. The implementation of this notion in the elaboration of nanocomplexes for the targeted delivery of drugs is particularly appealing due to the unique stimuli responsiveness of host–guest interactions [44,45]. Recently, Zhang and coworkers reported a superamphiphile construct encompassing a polyethylene glycol-tethered lactobionic acid-βCD conjugate (LA-PEG-βCD) and a benzimidazole-modified doxorubicine (BM-DOX) as the hydrophilic and hydrophobic constituents respectively, intended for the treatment of hepatocellular carcinoma (HCC) [46]. LA is a ligand of the galactose-specific asialoglycoprotein receptor (ASGPR) at the surface of liver cancer cells. The BM module fits well in the cavity of βCD: it was connected to the anticancer drug DOX through an acid-labile hydrazone functionality that was expected to be cleaved in the intracellular acidic environment with rapid release of DOX from the prodrug. In aqueous media, host–guest association between BM and βCD brought together the targeted supramolecular prodrug (TSPD), which spontaneously self-assembled into spherical, biocompatible nanoparticles that exhibited a pH-dependent drug release profile and excellent effects on HCC tumor cell growth (Figure 5).

**Figure 6:**
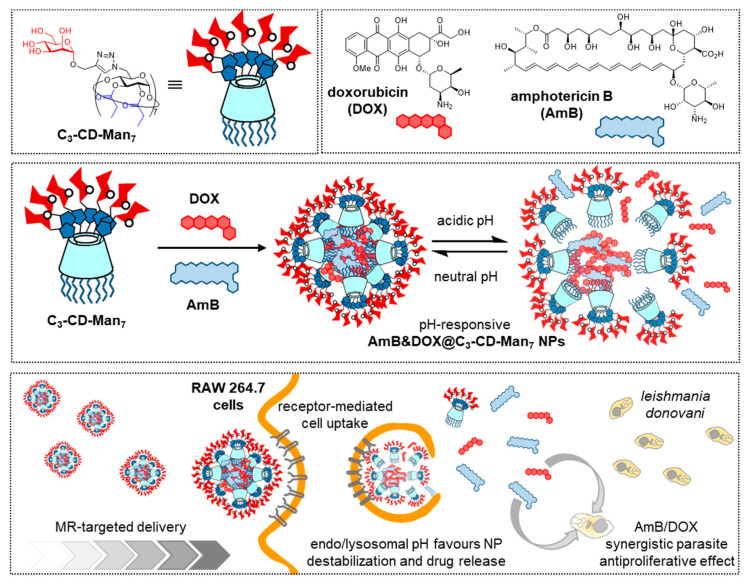
Structures of the GaCD derivative C_3_-CD-Man_7_, doxorubicin (DOX) and amphotericin B (AmB) (upper panel) and schematic representations of their co-assembly to afford pH-sensitive nanoparticles (Amb@DOX@C_3_-CD-Man_7_) (middle panel) and internalization in macrophages to treat visceral leishmaniasis (lower panel), as reported by Seeberger, Yin and coworkers [55,57]. GaCD, glycoamphiphilic cyclodextrin; Man, mannose; NP, nanoparticle.

The intrinsic two-face topology of CDs and the distinct reactivity of primary and secondary hydroxyls offers a convenient entry to covalent “classical” amphiphiles that can be tailored to self-assemble into different classes of aggregates (micelles, vesicles, nanospheres, nanocapsules) [17,47,48,49]. If the hydrophilic face in the amphiphilic CD is glyco-coated (glycoamphiphilic CDs; GaCDs), the corresponding self-assemblies will expose the glycan appendages to the bulk, available for their participation in biomolecular recognition processes. The interest of such systems for targeted drug delivery is obvious. Early work in the field was conducted by the groups of Nishimura, Darcy and Mazzaglia [50,51,52,53,54] and has already been reviewed [23]. Recent contributions by Seeberger, Yin and coworkers focus on the optimization of amphiphilic mannosylated βCD derivatives in terms of self-assembling properties and stability of the resulting mannose-functionalized nanoparticles [55]. The authors encountered that installation of seven mannosyl residues at the primary rim through copper(II)-catalyzed azide-alkyne cycloaddition (CuAAC) “click” coupling [56] and propanoyl ester groups at the fourteen secondary hydroxyls afforded conjugates (C_3_-CD-Man_7_) that self-assembled in water. In this manner, they obtained spherical nanoparticles with an average diameter of 45 nm (TEM), an average hydrodynamic diameter of 112 nm (DLS), low polydispersity index (PDI, 0.109) and high stability under physiological conditions. C_3_-CD-Man_7_ nanoparticles were able to encapsulate up to 12% of DOX, and the DOX-loaded system (DOX@C_3_-CD-Man_7_) efficiently delivered the drug to MR overexpressing MDA-MB-231 breast cancer cells in vitro and in vivo. The targeted drug delivery capabilities of C_3_-CD-Man_7_ were further confirmed for the treatment of visceral leishmaniasis (VL) [57]. The resulting AmB&DOX@C_3_-CD-Man_7_ nanomicelles were selectively taken up by RAW264.7 macrophages, with high expression of MR, and their therapeutic effect was established in vitro in RAW264.7 cells infected with *L. donovani* parasites. After treatment with AmB&DOX@C_3_-CD-Man_7_, almost no parasite was detected, supporting that the formulation is a promising solution for VL therapy (Figure 6).

## 4. Tailoring Glycopolymer Topology by CD Inclusion-Promoted Macromolecular Folding and Self-Assembly

**Figure 7:**
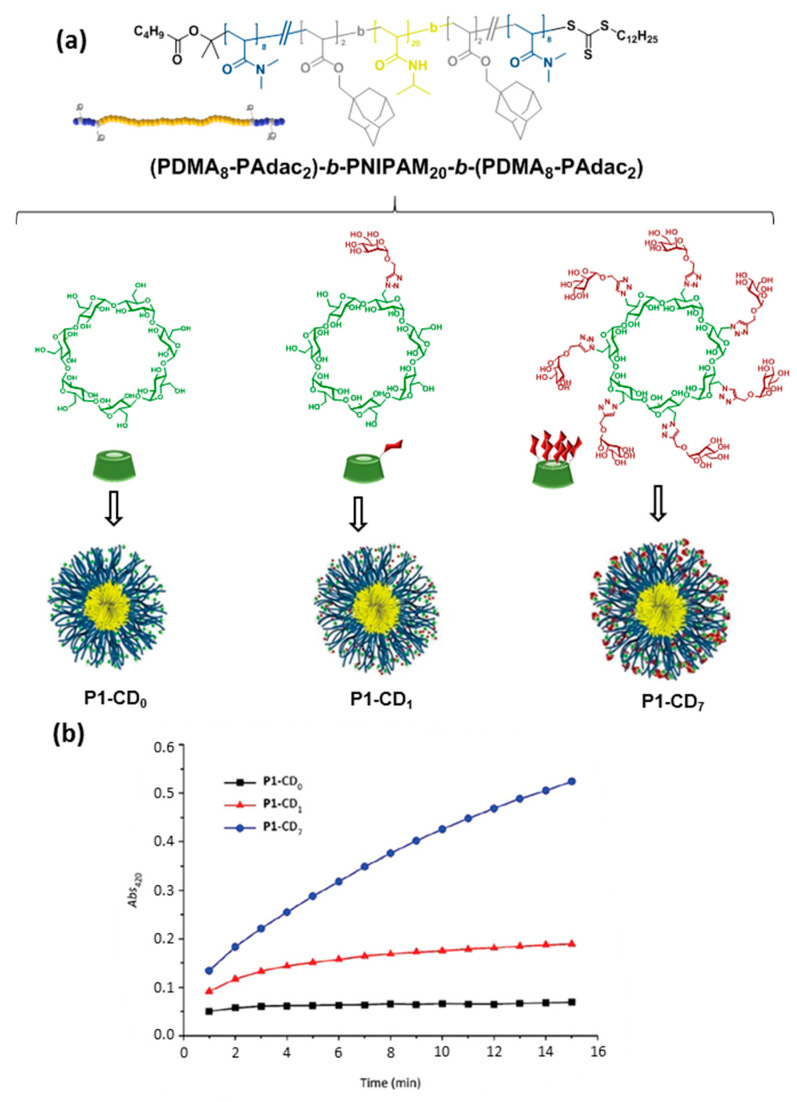
Average structure of the triblock copolymer (P1) and structures of the βCD derivatives used by Bercer and coworkers [61] to self-assemble glycomicelles trough equimolecular mixing in water (**a**) and turbidity plots obtained upon mixing with concanavalin A (ConA) at 40 °C (**b**). Adapted with permission from Reference [61]. Copyright 2016 Royal Society of Chemistry. PDMA, poly(*N*,*N*-dimethylacrylamide); PNIPAM, poly(*N*-isopropyl acrylamide); PAdac, poly(adamantane-acrylate).

The inclusion complexation between CDs, comprising CD units incorporated in polymers, and various guests has been extensively investigated in supramolecular chemistry [18]. However, although CD-containing polymers have been used in pharmaceutical applications since the 1980’s [1,5,58,59], the profitable features of inclusion complexation did not sufficiently draw attention and therefore were not employed in macromolecular self-assembly until about the beginning of this century [60]. CDs are particularly valuable and extensively employed in tuning the amphiphilicity of macromolecules. For instance, if a fragment of a macromolecule containing a guest moiety is connected to CDs via inclusion complexation, this area will become more hydrophilic and thus the amphiphilicity of the complete macromolecule as a whole is altered. This strategy allows to control the self-assembly and the morphology of the assemblies [15]. In 2015, Bercer and coworkers extended the inclusion complexation-driven macromolecule-associated micellization strategy to the use of glyco-coated CDs [61]. The authors prepared an ABA triblock copolymer architecture having precisely positioned random poly(*N*,*N*-dimethylacrylamide) (PDMA)/poly(adamantane-acrylate) (PAdac) blocks within the first and the third blocks, while the middle block consisted of thermo-responsive poly(*N*-isopropylacrylamide) (PNIPAM) that enables the formation of a self-assembled micellar structure above the cloud point. The Ad-functionalized thermo-responsive triblock copolymer provided supramolecular host–guest interactions with βCD as well as mono- and heptamannosylated βCD derivatives (CD-Man_1_ and CD-Man_7_). The resulting supramolecular amphiphilic macromolecule formed stable micelles in water as was clearly revealed by DLS. Quantitative concanavalin A (ConA; a mannose-specific plant lectin) precipitation assays demonstrated the accessibility of the mannosyl residues and their multivalent presentation at the surface of the assemblies, even in the case of the monomannosylated glycoCD, since lectin precipitation requires high carbohydrate density. Altogether, the results validated the Ad/βCD host–guest tactic for careful fine-tuning of the interaction between lectins and supramolecular glycopolymers (Figure 7).

**Figure 8:**
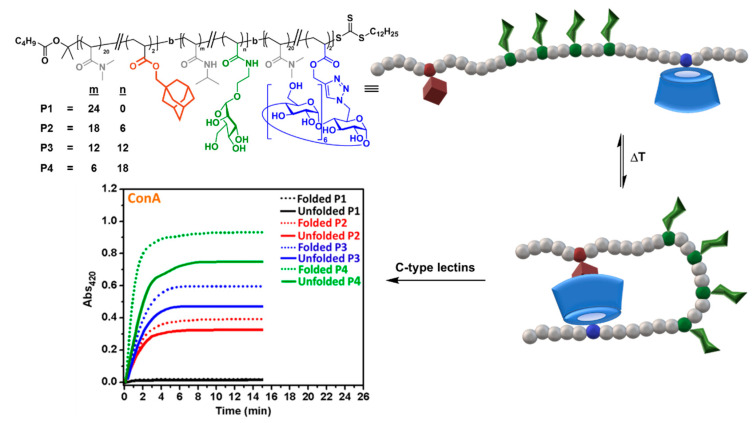
Triblock glycocopolymers bearing βCD units designed by Bercer and coworkers [63] to modulate the C-type lectin binding properties through intramolecular host–guest control of the folding state. Adapted with permission from Reference [63], https://pubs.acs.org/doi/abs/10.1021/acs.biomac.8b00600. Copyright 2018 American Chemical Society. ConA, concanavalin A.

The alliance between CD supramolecular chemistry and living polymerization has been particularly fruitful at providing fascinating polymer materials [62]. Bercer and coworkers applied one of the most popular of such reactions, reversible addition−fragmentation chain transfer (RAFT), to create triblock glycocopolymers bearing βCD, Ad and mannose residues, combined with PDMA, in different blocks [63]. At sufficiently high dilution in aqueous solution, intermolecular host–guest interactions were prevented and only single-chain folding, promoted by intramolecular CD/Ad interactions, were observable by bidimensional nuclear Overhauser effect spectroscopy (2D NOESY) nuclear magnetic resonance (NMR) and dynamic light scattering (DLS). Folded glycopolymers were unfolded at high temperatures and also by addition of the competitive guest molecule 1-adamantylamine hydrochloride. Interestingly, the folding state strongly affected the binding affinity towards the mannose-specific ConA, DC-SIGN and DC-SIGN-related (DC-SIGNR) lectins, as determined by turbidimetry and surface plasmon resonance (SPR) assays, providing direct evidence of the critical importance of controlling the secondary structures of glycopolymers for biological applications (Figure 8).

## 5. GlycoCD Rotaxanation and Self-Aggregation Strategies

**Figure 9:**
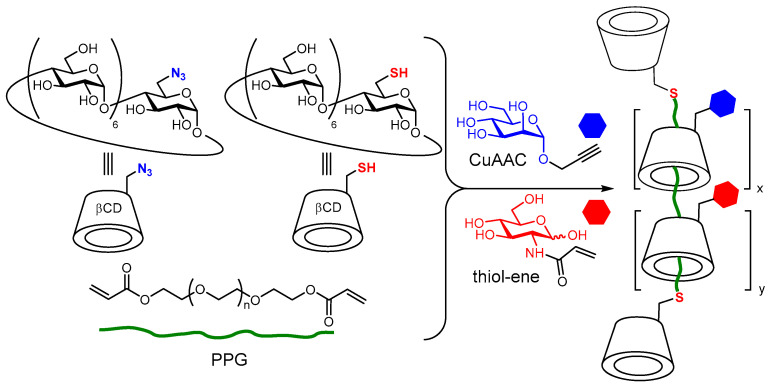
One-pot multicomponent synthesis of polyrotaxane-based heteroglycopolymers developed by Gao, Chen and coworkers [68]. CuAAC, copper(II) azide-alkyne cycloaddition; PPG, polypropylene glycol.

Treading glycoCD “beads” onto linear polymeric “strings” affords supramolecular dynamic systems, namely polyrotaxanes (if the polymer chain bears capping end-groups) or pseudopolyrotaxanes (if the capping groups are missing), where the glycoCD components can spin around the axes of the polymer as well as move back and forth along the polymer chain. Multivalent interactions with lectin partners can thus be maximized. Pioneering work in this area was conducted by the groups of Stoddart [64], Yui [65], Sasabe [66] and Fort [67] and has already been reviewed [23]. In 2017, Gao, Chen and coworkers proposed a one-pot multicomponent synthesis of pseudopolyrotaxane-based heteroglycopolymers anchored with different sugar units and fluorescent moieties via the combination of host–guest interaction, thiol-ene and copper(I)-catalyzed click chemistry in water [68]. The interest in multivalent (nano)systems exposing more than just a single type of saccharide motif has greatly increased in the last years after the publication of a cumulative number of reports describing synergistic effects in lectin binding, a phenomenon termed heterocluster effect [69,70,71,72,73,74,75]. Differently from earlier work, here, the glycoCD elements are generated in situ: mono(C-6)-azido and mono(C-6)-thio βCD are combined with propargyl-armed mannose and *N*-acryloylglucosamine derivatives in the presence of linear chain polypropylene glycol (PPG) and Cu(I) [68]. In this manner, the conjugation reactions and the host–guest processes take place simultaneously. The authors additionally prepared the analogous heteroglycopolyrotaxanes from pre-synthesized mannosyl-βCD and glucosamine-βCD conjugates, using in this case *O*-acryloyl-terminated PPG that undergoes thiol-ene coupling with mono(C-6)-thio βCD. ConA binding studies and aggregation experiments with *Escherichia coli*, known to expose the mannose biding lectin FimH in the type 1 fimbriae, evidenced the existence of symbiotic relationships ascribable to the heterocluster effect (Figure 9).

**Figure 10:**
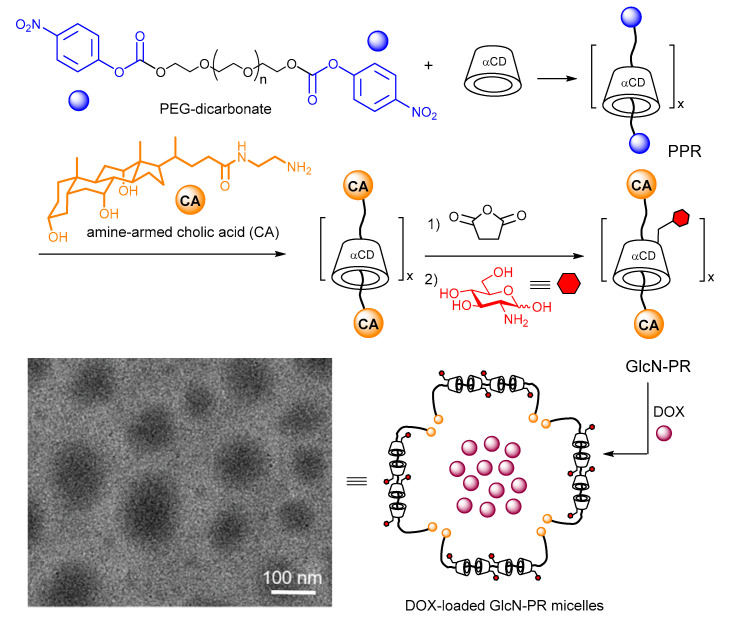
Polyrotaxane-based glycopolymers developed by Jia, Ren and coworkers [76] and a schematic representation of their self-assembly into doxorubicin-loaded micelles for the targeted delivery of the drug to tumor cells. A representative transmission electron microscopy (TEM) micrograph of the later is also shown. Adapted with permission from Reference [76]. Copyright 2019 WILEY-VCH. PEG, polyethylene glycol; PPR, pseudo(polyrotaxane); GlcN-PR, 2-amino-2-deoxy-β-D-glucopyranose-appended αCD-based polyrotaxane; DOX, doxorubicin.

Jia, Ren and coworkers reported an alternative post-functionalization strategy for glycopolyrotaxane synthesis, whereby αCD was first threaded onto *p*-nitrophenyl carbonate-ended PEG chains followed by amide-forming capping with an amine-equipped bile acid derivative. The primary positions of the αCD beads were next succinylated and the resulting interlocked poly(carboxylic acid) was subsequently conjugated with glucosamine. The glyco-αCD/PEG polyrotaxane thus obtained formed micelles in aqueous solution that exhibited remarkable doxorubicin-loading capabilities and selectively delivered the drug to mouse 4T1 breast cancer cells and not to normal NIH3T3 cells [76] (Figure 10).

**Figure 11:**
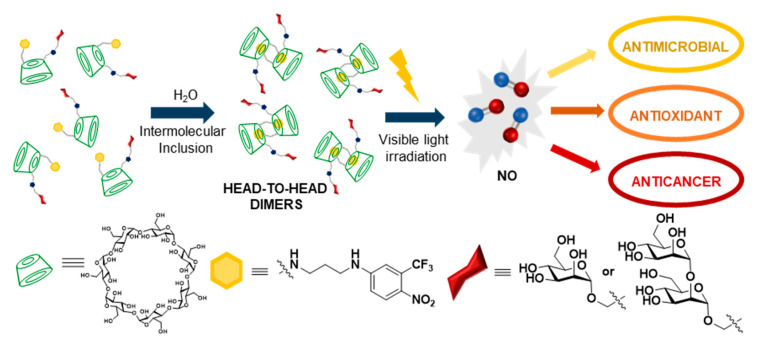
Structures and schematic representation of the functional supramolecular glycosystems developed by Vargas-Berenguel and coworkers for targeted nitric oxide-based therapies [80]. NO, nitric oxide.

CD derivatives bearing substituents that fit the cyclooligosaccharide cavity can undergo self-inclusion or intermolecular inclusion phenomena. If reciprocal, the second process affords supramolecular homodimers, whereas if it propagates, poly-pseudo[2]rotaxane-type supramolecular oligomers can form [77,78,79]. Vargas-Berenguel and coworkers explored the possibility of exploiting this approach for the assembly of functional glycosystems from βCD precursors randomly substituted at the primary positions with mannosyl or α(1→2)-mannobiosyl residues (through CuAAC click conjugation) and 4-nitro-3-(trifluoromethyl)aniline moieties (through propylenediamine bridging) [80]. The latter is a nitric oxide (NO) photodonor (NOPD) that offers the possibility to deliver NO with high spatiotemporal control, thus favoring reducing side-effects and improving therapeutic outputs, e.g., for antimicrobial, antioxidant or anticancer applications. From hydrodynamic diameters (d_H_) measurements, the authors concluded that head-to-head dimeric species are predominant in aqueous solution, which translates into a two-fold increase in valency and a significant multivalent effect, as determined for ConA lectin binding. The dimers also displayed increased NO photodelivery efficiency upon irradiation with visible light, which supports their potential for targeted NO-based therapies (Figure 11).

## 6. Biomacromolecule-Templated Formation of Functional GlycoCD Nanoassemblies

**Figure 12:**
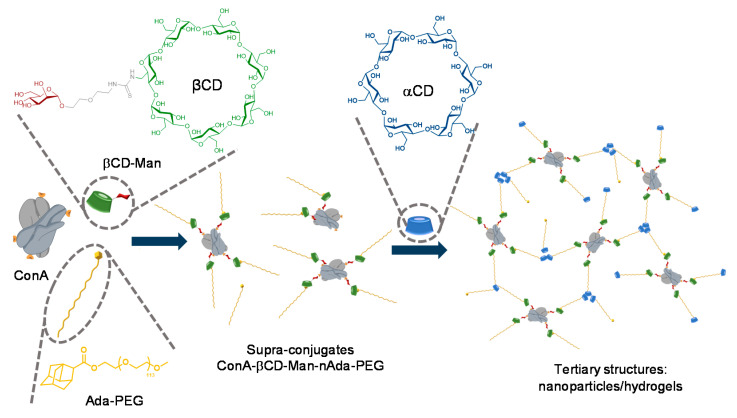
Molecular structures and schematic illustrations of the mannosylated βCD derivative βCD-Man, the adamantane-equipped poly(ethylene glycol) derivative Ada-PEG, concanavalin A (ConA) and αCD, and of their combination to obtain supramolecular nanoparticles and hydrogels, as reported by Chen and coworkers [82]. CD, cyclodextrin; Man. Mannose; Ada-PEG, adamantane-equipped poly(ethylene glycol), ConA, concanavalin A.

Besides polymers, many kinds of biomolecules, including proteins and nucleic acids, also bear great potential as building blocks to form hierarchical glyconanomaterials by controllable co-assembly with glycocyclodextrin partners. In order to achieve specific functionalities, a key issue is to effectively adjust the supramolecular interactions to form the desired structures. Differently from synthetic polymers, for which the incorporation of ad hoc motifs enabling multiple host–guest phenomena can be easily implemented, programming cyclodextrin–biomolecule associations requires emulating biomimetic mechanisms relying primarily in hydrogen bonding, electrostatic, polar, hydrophobic or ligand–receptor interactions [81]. Such an approach implies the incorporation of functional elements in the CD platform, allowing those processes to occur in a predetermined manner. Eventually, the CD macro-ring can be additionally engaged in second-level supramolecular events, offering excellent opportunities for finely tuning glyconanomaterial properties. Chen and coworkers [82] smartly illustrated this concept by exploiting the ability of the mannosylated βCD derivative βCD-Man to crosslink ConA and adamantane-equipped poly(ethylene glycol) chains (Ada-PEG). The ability of the βCD cavity to host the adamantane fragment with high affinity was found to be unaffected by the recognition of the sugar ligand by the lectin. Similarly, the behavior of mannose as a ligand in biological interactions was not altered by the presence of the supramolecular host, confirming the dual molecular recognition abilities of βCD-Man. ConA is a tetramer at neutral pH, each monomer bearing a single mannose binding site. Given that the binding constant for the mannose-ConA association is rather weak (*K*_a_ ≈ 10^3^ M^−1^), unless multivalently presented, the number of binding sites occupied in βCD-Man/Ada-PEG/ConA supra-conjugates was strongly dependent on the concentration of the constitutive building blocks, which was used by the authors to regulate the assembly behavior. A third supramolecular level was next implemented by adding αCD, which is known to form pseudo-polyrotaxanes upon threading in PEG chains, thereby driving further assembly of the conjugates into large objects. By modulating the different supramolecular phenomena at play, diverse nanoobjects, from nanoparticles to high-strength hydrogel, could be engineered (Figure 12).

**Figure 13:**
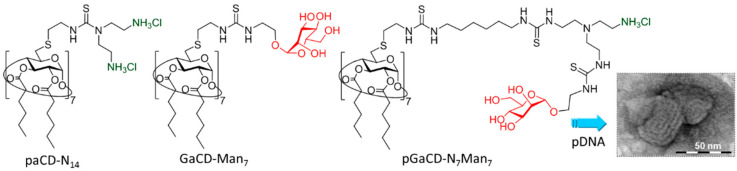
Structures of the polycationic amphiphilic βCD derivative paCD-N_14_ (14 cationizable primary amines), the glycoamphiphilic βCD derivative GaCD-Man_7_ (neutral; 7 mannosyl residues) and the polycationic glycoamphiphilic βCD derivative (7 cationizable primary amines and 7 mannosyl residues) reported by García Fernández and coworkers [156]. A representative image at high magnification of the CDplexes obtained from the latter and the luciferase-encoding plasmid DNA (pDNA) pTG11236 (5739 bp) is also shown. Adapted with permission from Reference [156]. Copyright 2011 Elsevier.

Nucleic acid molecules are polyanions in aqueous solution and thus electrostatically interact with various types of polycations. The resulting polyelectrolyte complexes have found application in gene delivery technology [83,84]. Most nonviral nucleid acid carriers (vectors) reported are polymeric, conformationally undefined or complex nanoparticulate materials [85]. In the last decade, however, multivalent polycations based on molecular systems emerged as a promising alternative [86,87,88,89,90,91,92,93,94,95,96]. Polycationic cyclodextrins have become the iconic representatives in this category [5,97,98,99,100,101,102,103,104]. The efficiency in compacting nucleic acids into nanocomplexes (so-called CDplexes) capable of mediating cell entry and subsequent expression of the nucleic acid anticipated activity (transfection) was significantly enhanced for prototypes that can additionally establish cyclodextrin–cyclodextrin interactions through either hydrophobic [105,106,107,108,109,110,111,112,113,114,115,116,117,118,119,120,121,122,123,124,125,126,127,128,129,130,131,132,133], host–guest [79,134] or aromatic–aromatic contacts [135,136,137,138,139,140,141,142,143,144,145,146]. As a logical extension, the formulation of CDplexes exposing biorecognizable ligands for the purpose of site-specific gene delivery has been proposed [147,148,149,150,151,152,153,154,155], including the elaboration of carbohydrate decorated CDplexes (glycoCDplexes) conceived for cell receptor glycotargeting [3]. In a first attempt, García Fernández and coworkers prepared three-component supramolecular assemblies from a multi-head/multi-tail polycationic amphiphilic CD displaying 14 primary amino groups (paCD-N_14_), a glycoamphiphilic CD (GaCD) bearing mannosyl residues at the seven primary βCD positions (GaCD-Man_7_) and a luciferase-encoding plasmid DNA (pDNA; pTG11236, 5739 bp) (Figure 13) [156]. paCD-N_14_ was known to form CDplexes that efficiently mediated transfection in several cell lines. Disappointingly, the presence of the neutral GaCD-Man_7_ component, even at low (5–10%) proportion, resulted in nanoparticles that failed to protect pDNA from the environment. The authors ascribed the negative result to a mismatching effect of segregated cationic and neutral microdomains upon pDNA templation, leading to CDplex destabilization, and proposed a new vector design to overcome this issue: polycationic glycoamphiphilic CDs (pGaCDs) presenting regular arrangements of glyco-cationic moieties. To test this conception, a compound bearing the cationic centers (seven) and the mannosyl moieties (seven) at distinct branches in a perfectly monodisperse *C*_7_ symmetrical dendroidal architecture, namely pGaCD-N_7_Man_7_, was synthesized. Gratifyingly, pGaCD-N_7_Man_7_ formed homogeneous distributions of small-size glycoCDplexes with pDNA (80 ± 35 nm, as determined by DLS) that fully prevented the nucleic acid cargo from degradation by nucleases. TEM micrographs revealed a snake-like ultrastructure probably arising from alternating packing of electron-dense (pDNA strains) and electron-deficient (lamellar pGaCD arrangements) regions (Figure 13). The mannosylated nanocomplexes efficiently recognized the mannose-specific lectins ConA and human macrophage mannose receptor (hMMR), as ascertained by the enzyme-linked lectin assay (ELLA) [157,158,159,160]. Most notably, they promoted specific internalization and transfection in RAW264.7 (mouse leukemic monocyte macrophage) cells, known to express mannose receptors involved in receptor-mediated endocytosis at the cytoplasmic membrane.

**Figure 14:**
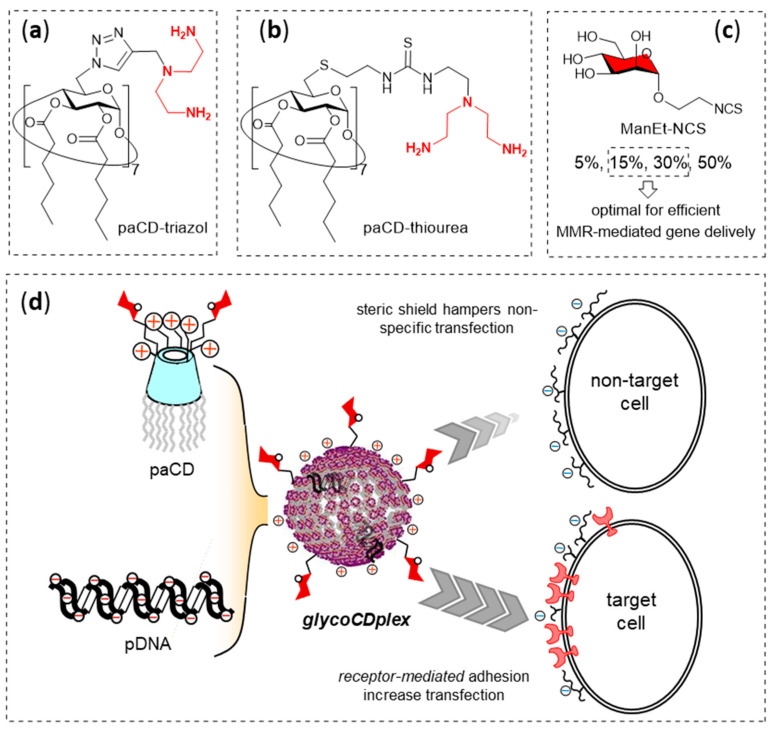
Structures of the paCD-triazol (**a**), paCD-thiourea (**b**) and ManEt-NCS (**c**) precursors used by Di Giorgio, Benito and coworkers [164] to prepare pGaCDs with variable proportions of mannosyl motifs, and a schematic representation of their co-assembly with pDNA to form glycoCDplexes that selectively promoted receptor (MMR)-mediated transfection of the targeted cells (macrophages) (**d**). Adapted with permission from Reference [164]. Copyright 2015 Royal Society of Chemistry. paCD, polycationic amphiphilic cyclodextrin; Man, mannose; pGaCDs, polycationinc glycoamphiphlic cyclodextrins; MMR, macrophage mannose receptor.

GlycoCDplexes formed upon nucleic acid templation of pGaCDs shall expose multiple copies of the saccharide motif at the periphery. In other words, they will bear intrinsic multivalency, an important requisite for the recognition of functional glyconano(bio)materials by cognate lectins [161,162,163]. In principle, the ligand surface density will increase with the pGaCD valency, which would be expected to translate into enhanced lectin binding avidities. Yet, the presence of the sugar residues in the vector architecture may negatively affect the nucleic acid complexation/nanocondensation process by shielding the cationic centers or by decreasing their number (e.g., if their incorporation involves cancelling pre-existing amino groups). Harmonizing nucleic acid and lectin binding properties through careful pGaCD design is therefore essential in the context of nucleic acid glycotargeting strategies. Di Giorgio, Benito and coworkers addressed this issue for the particular case of macrophage-directed gene delivery [164]. The authors selected two lead paCDs featuring 14 primary and 7 tertiary amino groups at the primary face and 14 hexanoyl tails at the secondary rim, but differing in either the presence of 1,2,3-triazol (paCD-triazol; Figure 14a) or thiourea linkers (paCD-thiourea; Figure 14b). Both the paCD-triazol and paCD-thiourea precursors were reacted with 2-isothiocyanatoethyl α-D-mannopyranoside (ManEt-NCS; Figure 14c) in variable proportions, in order to achieve conjugates with 5%, 15%, 30% and 50% of the primary amino groups transformed into mannopyranosylethylthioureido segments. As expected, the capability to neutralize and fully protect pDNA decreased with the mannosylation degree. Conversely, glycoCDplexes formulated with the more heavily mannosylated vectors showed higher stability in saline medium (150 mM), probably by endowing the nanoparticles with a thicker hydration shell that prevents non-specific aggregation phenomena. Most importantly, mannosylated CDplexes exhibited remarkably alveolar peritoneal macrophage (mice) adhesion abilities. In vitro experiments using MMR-positive mouse leukemic monocyte macrophages (RAW264.7), MMR-devoid embryonic murine hepatocytes (BNL-CL2) and African green monkey kidney fibroblasts (COS-7) confirmed that mannosylation results in notable transfection selectivity enhancements towards the macrophage cell line (Figure 14d). Taking all data together, 30% and 15% mannosyl loadings were determined to be optimal in the triazol and thiourea series respectively, to achieve glycoCDplexes with high macrophage transfection levels and minimal off-target transfection in other cells.

**Figure 15:**
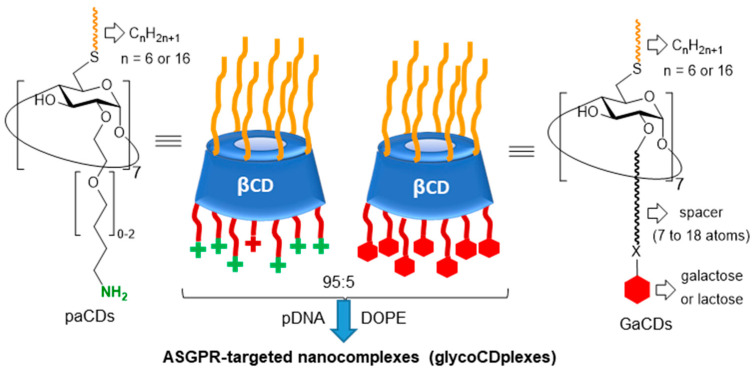
Structures of the polycationic amphiphilic CDs (paCDs) and glycoamphiphilic CDs (GaCDs) used by O’Driscoll and coworkers [165] for the formulation of pDNA-templated nanocomplexes (glycoCDplexes) targeting the asialoglycoprotein receptor (ASGPR). DOPE, 1,2-dioleoyl-sn-glycero-3-phosphoethanolamine.

O’Driscoll and coworkers [165] prepared pDNA-templated glycoCDplexes combining a paCD [166] and a galactosylated or lactosylated neutral amphiphilic CD [51] in 95:5 (*w*/*w*) relative proportion, in view of targeting the galactose/galactosamine-specific asialoglycoprotein receptor (ASGPR) at the surface of parenchymal hepatocytes (Figure 15). The authors monitored the transfection efficiency in human cellular ASGPR-positive hepatocarcinoma Hep-G2 cells and found that statistically significant enhancements, as compared with non-targeted formulations, were achieved only when the helper lipid 1,2-dioleoyl-sn-glycero-3-phosphoethanolamine (DOPE) was included in the formulation. Confocal microscopy studies revealed that the incorporation of DOPE in targeted systems did not lead to higher cell uptakes of galactosylated as compared with non-galactosylated nanocomplexes but resulted in a much faster endosomal escape of the glycotargeted nanocomplexes upon receptor-mediated endocytosis.

**Figure 16:**
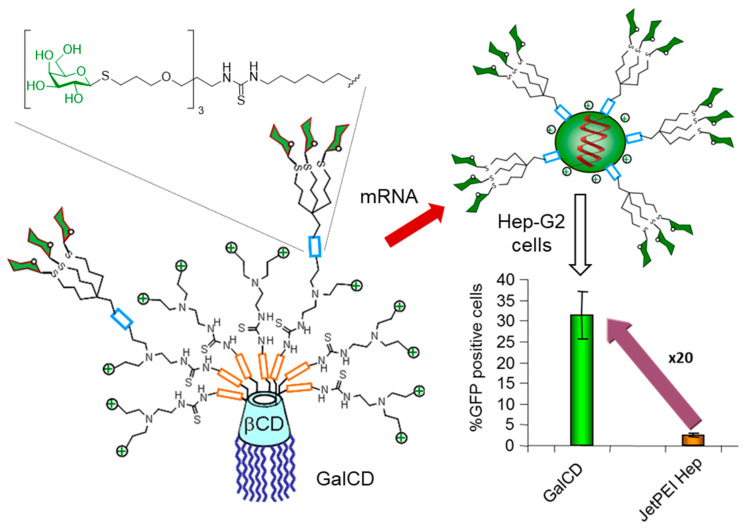
Schematic representation of the statistically galactosylated paCD (GalCD) vectors used by Rejman and coworkers to formulate glycoCDplexes with mRNA for the ASGPR-mediated transfection of hepatocytes [167].

To avoid the need for using DOPE-containing polyformulations and the high synthetic cost associated to fully regular, diasteremerically pure pGaCD vectors, Rejman and coworkers proposed an alternative strategy consisting in the statistic conjugation of paCDs with glycodendrons [167]. Indeed, CD-glycodendrimer constructs have shown high promise for lectin-targeted, site-specific drug delivery [35,158,168,169]. The notion behind this is that biologically useful multivalent presentations can be achieved in this manner with no need for sacrificing many cationic centers in the conjugation step, thus preserving the nucleic acid nanocomplexation properties intact. As a proof of concept, the authors incorporated a trivalent galactosyl dendron, a good ligand for the ASGPR, onto a paCD that exhibited good gene vector properties, in 10% or 12% proportions relative to the peripheral amines. Both conjugates were able to complex a green fluorescent protein (GFP)-encoding pDNA, but puzzlingly, the resulting glycoCDplexes turned out to be inefficient at transfecting Hep-G2 cells. Specific tests were conducted to ascertain at what stage in the process from uptake to translation the pDNA-glyoCDplexes failed. Neither internalization nor endosomal scape or pDNA release were found to be problematic, with the ensemble of results suggesting that translocation of the pDNA into the nucleus represented the main obstacle. Indeed, replacing pDNA into messenger RNA (mRNA), which does not need to reach the nucleus to express the encoded protein, resulted in outstandingly high transfection levels, much higher than those achieved with the commercial polymeric vector poly(ethyleneimine)-hepatocyte (jetPEI-Hepatocyte) (Figure 16). Blocking the ASGPR with anti-ASGPR antibodies cancelled transfection, demonstrating that the galactosyl-CDplexes enter the cells through ASGPR-mediated endocytosis.

**Figure 17:**
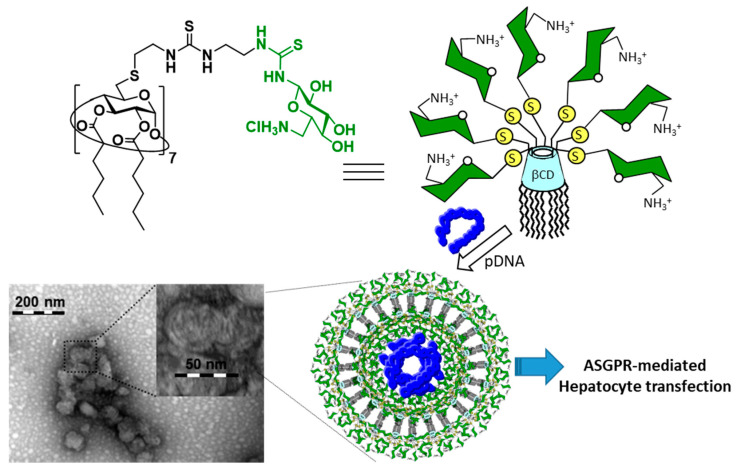
Structure of the 6-amino-6-deoxy β-D-glucopyranosylthioureido/βCD conjugate prepared by Jiménez Blanco, Di Giorgio and coworkers, and an illustration of their co-assembly with pDNA to form nanocomplexes that promoted ASGPR-mediated transfection of hepatocytes [170,171]. A representative TEM micrograph of the nanocomplexes is also shown. Adapted with permission from Reference [170]. Copyright Centre National de la Recherche Scientifique (CNRS) and Royal Society of Chemistry.

Jiménez Blanco, Di Giorgio and coworkers proposed an alternative pGaCD vector design that displayed aminoglucosyl units attached at all primary positions of the per(*O*-2,*O*-3)-hexanoylated βCD scaffold, instead of bearing amine groups and sugar ligands at separate branches [170]. They encountered that the self-assembling and transfection aptitude of these compounds were very sensitive to the aminoglycoside structure: 6-amino-6-deoxy and 2-amino-2-deoxy-β-D-glucopyranosylthioureido conjugates readily formed glycoCDplexes (75–100 nm hydrodynamic diameter) in the presence of pDNA, but only the first ones were efficient at promoting transfection in COS-7 cells. A homologous heptaconjugate exposing 2,6-diamino-2,6-dideoxyglucoside motifs formed a solid precipitate in the presence of pDNA and was not further pursued. Most interestingly, the 6-amino-6-deoxyglucoside pGaCD was found to selectively bind the galactose-specific lectin peanut agglutinine (PNA) and preferentially internalize BNL-CL2 hepatocytes by ASGPR-mediated endocytosis [171], highlighting this iminosugar as a dual nucleic acid/lectin receptor binder when presented in multivalent form (Figure 17).

**Figure 18:**
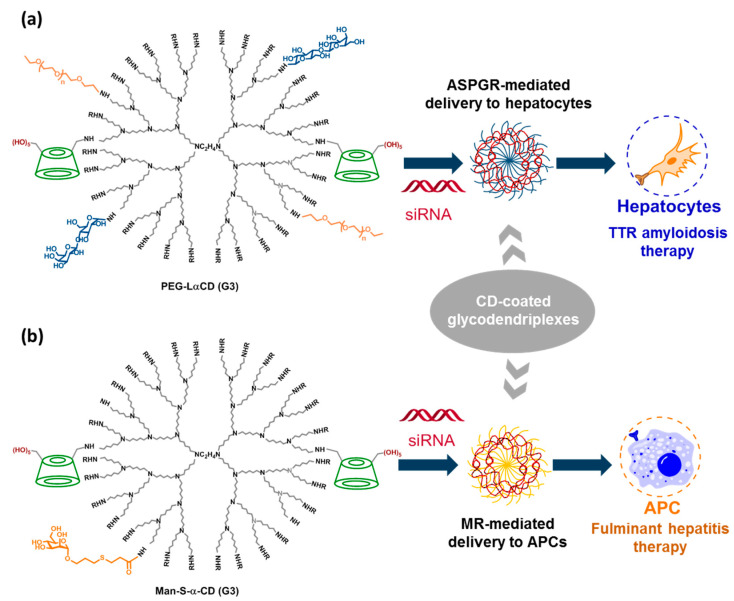
Structures of the lactosylated (**a**) and thiomannosylated (**b**) αCD-coated glycodendrimers prepared by Arima and coworkers and a schematic representation of their co-assembly with siRNA to form dendriplexes targeted to hepatocytes or antigen-presenting cells (APCs) respectively, for the treatment of transthyretin-related (TTR) amyloidosis [176] or fulminant hepatitis [177].

Uekama, Arima and coworkers discovered that poly(amidoamine) (PAMAM) dendrimers functionalized with αCD (αCDEs) formed nanocomplexes with nucleic acids (dendriplexes) with very high transfection capabilities. The increase in efficiency at delivering the cargo to target cells was ascribed to an enhanced endosomal escaping ability through the cooperative action of a proton sponge effect of PAMAM dendrimer and the inclusion ability of αCD towards phospholipids in endosomes [172,173]. A degree of substitution (DS) of 2.4 was revealed as optimal. By appending different glycoligands to remaining peripheral amine groups in the dendrimer, they engineered glyco-αCDEs that in the presence of pDNA or siRNA, formed glycodendriplexes specifically targeting complementary lectin receptors. Early work from this group has already been reviewed [97]. Recent versions of the general prototype include lactosyl- and mannosyl-αCDEs (generation 3, G3) for specific siRNA delivery to hepatocytes [174] and antigen-presenting cells (APCs) [175], respectively. The first ones were also decorated with PEG-chains (PEG-LαCD) and have shown promise in transthyretin-related (TTR) amyloidosis therapy [176], whereas the second (Man-S-αCD) showed potential for the treatment of fulminant hepatitis [177] (Figure 18).

## 7. Host–Guest Mediated Glyco-Coating of Self-Assembled CD Nanoparticles

**Figure 19:**
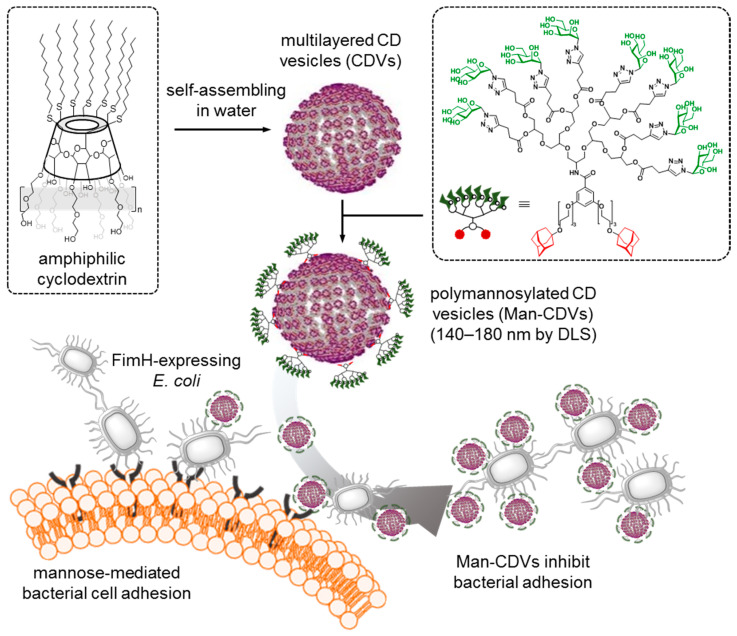
Illustration of the strategy developed by Ravoo and coworkers for the preparation of self-assembled CD-based vesicles (CDVs), their coating with mannosyl dendrons through host–guest interactions and the potential of the resulting Man-CDVs in anti-adhesive therapy against uropathogenic FimH-expressing *E. coli* [184].

The micellar or vesicular supramolecular materials assembled from amphiphilic CD molecules are intrinsically multicompartmental. Provided that access to the cavity of the individual cyclooligosaccharides at the surface of the aggregates is not blocked, they can be exploited as anchoring centers for functional moieties equipped with appropriate guest motifs. Based on these grounds, Ravoo and coworkers developed a host–guest approach to glycosylate the external surface of cyclodextrin vesicles (CDVs) [178,179]. A main advantage is that given the dynamic character of the interactions at play, the resulting glyco-CDVs better imitate the fluidity of the cell membrane as compared with nanosystems built from covalent glycoCDs. The vesicular nanoplatform was obtained from CD precursors where all primary hydroxyls have been replaced by dodecylthio tails and some of the secondary hydroxyls, mainly O-2-positions, are etherified with ethylene carbonate. The latter reaction results in the installation of oligo(ethylenglycol) chains, which on average are diethylenglycol residues, thereby creating a hydrophilic domain [180]. In spite of bearing a certain polydispersity, the self-assembled CDVs (average hydrodynamic diameter ≈ 120–150 nm) engineered from these CDs are very stable and behave as host membranes [181]. βCD-based CDVs and Ad-armed glycoconjugates have been most often employed to formulate glyco-CDVs [182], but other high-affinity guest motifs have also been explored, such as diamantane or triamantane derivatives. Interestingly, mannopyranosyl glycosides bearing the later diamandoids were efficiently fixed onto βCD as well as γCD CDVs, affording multivalent mannosyl-CDVs capable of recognizing mannose-specific receptors, as illustrated by their ability to agglutinate ConA lectin [183]. In the case of βCD CDVs, using bipodal adamantane branches as anchoring elements has proven particularly convenient, enabling the efficient conjugation with single mannosyl ligands but also with up to octavalent mannosyl glycodendrons. Such high-valent mannosyl-CDVs (Man-CDVs) proved very efficient at disturbing the interaction between a FimH lectin-expressing uropathogenic *E. coli* strain (ORN178) and the human uroepithelial cell line RT-4 [184] (Figure 19).

**Figure 20:**
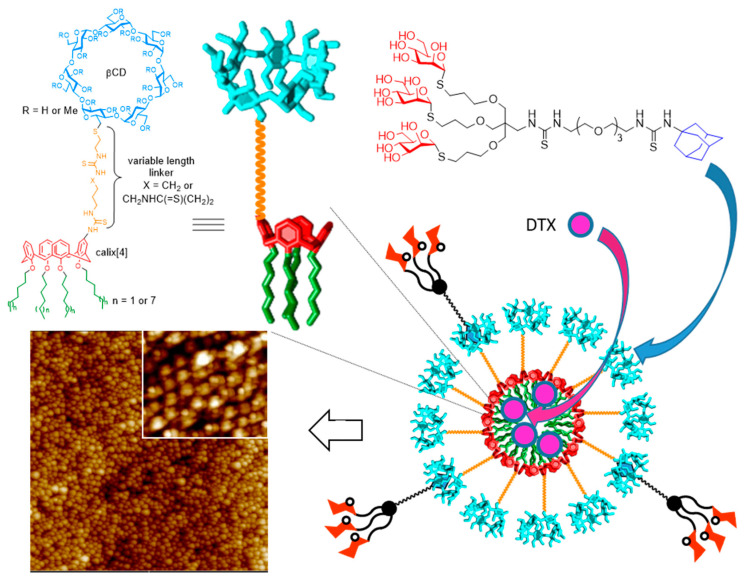
Illustration of the nanospheres (NSs) prepared by nanoprecipitation of βCD-calix[4]arene hybrids by Sansone, Casnati, García Fernández, Ceña and coworkers [185,186,187], their loading with anticancer drugs (e.g., docetaxel, DTX) and their host–guest decoration with glycoligands for targeted delivery. A representative tapping-mode atom force microscopy (AFM) image of the NSs (5 × 5 μM; insert 0.7 × 0.7 μM) is also shown. Adapted with permission from Reference [187]. Copyright Royal Society of Chemistry.

Sansone, Casnati, García Fernández, Ceña and coworkers have elaborated self-assembled CD nanospheres (NSs) and nanocapsules (NCs) from CD-calixarene heterodimers [185,186]. The basic prototype consists in a βCD module having either hydroxyl or methyl ether groups that is connected through a single primary position to a spacer element of variable length and nature, which also joins a calix[4]arene (calix[4]) component. The latter bear aliphatic chains etherifying the four phenolic oxygens at the lower rim, making it strongly hydrophobic. Upon nanoprecipitation in water in the absence or in the presence of a pharmaceutically acceptable oily additive, NSs (mean diameter 10–40 nm as determined by AFM and TEM) or NCs (50–100 nm) were obtained. In the NSs, the calix[4] hydrophobic matrix is surrounded by a CD-exposing hydrophilic shell, whereas in the NCs, the core is occupied by the oily medium. Both NSs and NCs showed high loading capacity towards the anticancer drugs docetaxel, temozolomide and combretastatin A-4 and high efficiency at delivering them to different cancer cells: LNCaP and PC3 prostate cancer, MCF-7 breast cancer, glioblastoma U87, HeLa cervical cancer and HT-29 colon cancer cells. As for the CDVs discussed above, the βCD units at the external shell can host the Ad part of glycodendron-Ad conjugates, imparting glycotargeting abilities. As a proof of concept, a trivalent mannosyl dendron was supramolecularly installed onto βCD-calix[4] NSs and the resulting glycol-NSs were shown to be specifically recognized by the MRR [187] (Figure 20).

## 8. Functional Glyconanoparticles through CD-Mediated Glyco-Coating Strategies

**Figure 21:**
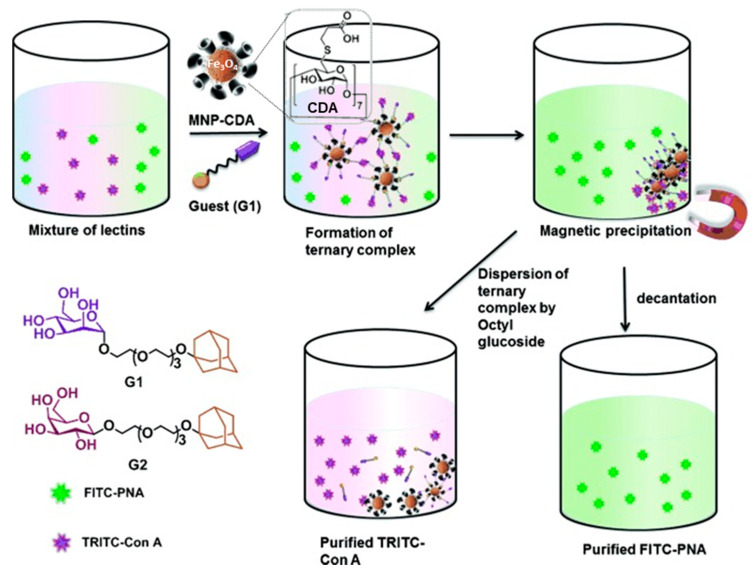
Illustration of the method for separation of proteins reported by Samanta and Ravoo [188] using the cross-linking and magnetic precipitation of magnetic nanoparticles modified with cyclodextrin-acid derivatives (MNP-CDA) and adamantane-armed glycoligands (G1 and G2). Reprinted with permission from Reference [188]. Copyright 2014 WILEY-VCH. FITC-PNA, fluorescein isothiocyanate-labelled peanut agglutinin; TRITC-ConA, tetramethylrhodamine-labelled concanavalin A.

Surface decoration with cyclodextrins is a general strategy to enhance the drug loading capabilities of nanocarriers and/or to supramolecularly anchor functional components for, e.g., site-specific delivery, visualization or sensing. The methodology is indeed analogous to that discussed above for self-assembled systems and relies in the formation of a dynamic external shell through multiple host–guest interactions between the CD units on the nanoparticle and bifunctional molecules consisting of a CD guest motif, a flexible tether and an outer probe moiety. If the latter is a glycoligand, the bifunctional molecules can behave as cross-linkers between the nanoparticle and specific lectins. The work reported by Samanta and Ravoo in 2014 on magnetic iron oxide (Fe_3_O_4_, γ-Fe_2_O_3_) nanoparticles (MNPs) capped with βCD and their use as support for selective supramolecular capture of lectins very didactically illustrates this concept [188]. The synthesis of the βCD-capped MNPs encompassed the preparation of bare MNPs by alkaline co-precipitation of Fe^II^ and Fe^III^ salts followed by ligand stabilization with heptakis(6-*S*-carboxylpropyl-6-thio-βCD) (CDA). The surface of the resulting CDA-modified MNPs (MNP-CDA) were next decorated with specific carbohydrates, namely mannose and lactose, by simply adding adamantane–carbohydrate conjugates to the nanoparticles. A multivalent presentation of the monosaccharide is thus generated that orthogonally cross-linked the mannose and galactose specific lectins ConA and PNA, respectively, allowing their efficient separation from mixtures of both proteins by magnetic precipitation. The pure lectins were finally detached from the ternary complexes by addition of an excess of the corresponding octyl glycoside. More recent work has shown that the reciprocal distribution of functional moieties, that is, the use of nanoparticles displaying adamantane elements in combination with biorecognizable glycoCDs, is also found as a viable alternative for lectin purification or bacterial capture [189,190] (Figure 21).

The above host–guest-based approach, in whatever of the two commented modalities (CD-coated nanoparticles in combination with glycoligand-equipped cross-linkers or CD guest-coated nanoparticles in combination with glycoCDs) is very versatile and has been successfully extended to the non-covalent peripheral functionalization of a variety of nanometric platforms with carbohydrates for analytical or biomedical applications. Examples on record include host–guest mannose- and galactose-modified quantum dots (glyco-QDs) for the optical detection of carbohydrate–protein interactions [191,192] and mannose-decorated mesoporous silica-coated gold nanorods [193] and graphene shits [194] for *E. coli* bacteria agglutination and killing.

## 9. Covalent Strategies to CD-Appended Glyconanomaterials

**Figure 22:**
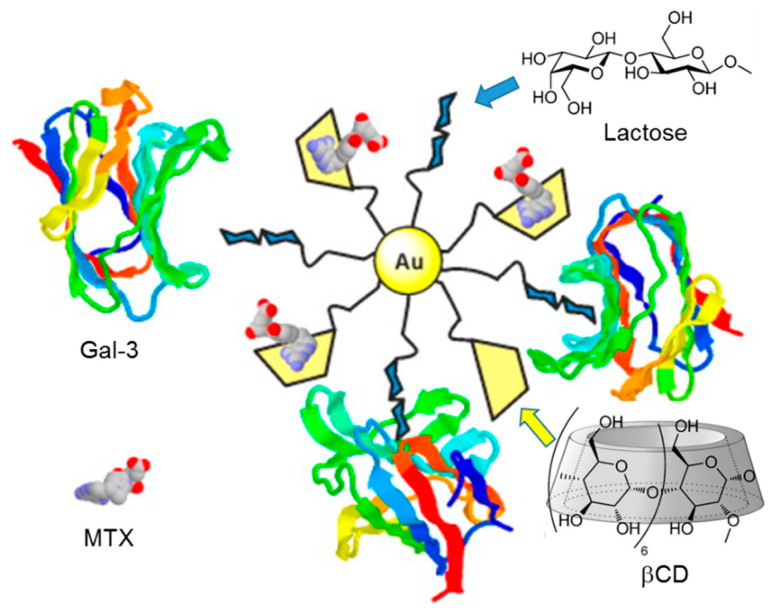
Illustration of the lactose- and βCD-decorated gold nanoparticles developed by Vargas-Berenguel and coworkers [195] for the selective delivery of methotrexate (MTX) to human tumors expressing galectin-3 (Gal-3). Adapted with permission from Reference [195]. Copyright 2014 American Chemical Society.

Coating a given nanoplatform with cyclodextrins and glycan moieties, or with a glycoCD conjugate, is probably the most obvious plan to engineer nanosystems endowed with inclusion capabilities and lectin recognition properties. In 2014, Vargas-Berenguel and coworkers put this approach at work to prepare dually functionalized gold nanoparticles (AuNPs) simultaneously bearing β-cyclodextrin and lactose appendages for the development of site-specific drug delivery systems [195]. The synthesis of the CD/lactose-coated AuNPs was achieved from CD and lactose disulfide precursors by the well-known ligand exchange strategy on citrate-stabilized AuNPs. The spacer arm combined a hydrophobic portion intended to provide stability to the self-assembled monolayer on the AuNP surface and a tetraethylenglycol segment to increase the biocompatibility and the solubility in water. The presence of the cyclodextrin units made possible the loading of the anticancer drug methotrexate (MTX) by forming stable inclusion complexes, whereas the multivalent presentation of lactosyl residues imparted binding abilities towards the β-D-galactose-recognizing lectins PNA and human galectin-3 (Gal-3). Gal-3 is well-known to be overexpressed in several human tumors and is therefore a biomedically relevant target, making such hybrid AuNPs promising nanocarriers for precision anticancer therapies (Figure 22).

**Figure 23:**
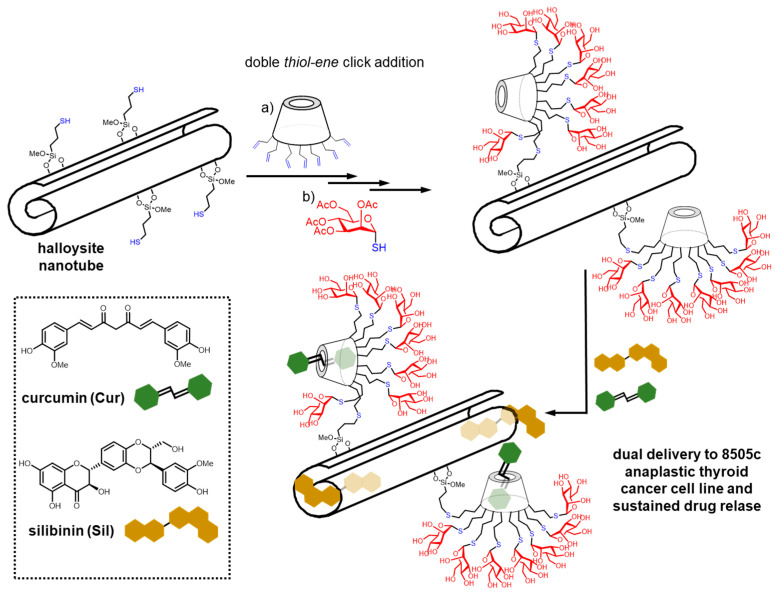
Structures and schematic representation of the strategy developed by Riela, Lazzala and coworkers to assemble hybrid glycoCD/halloysite nanotubes to achieve functional glyconanomaterials for the co-administration of curcumin (Cur) and silibinin (Sil) in thyroid cancer cells [196,197].

Riela, Lazzala and coworkers reported a new design of glycomaterials consisting of halloysite nanotubes (HNTs) coated with βCD-based glycoclusters [196]. Such hybrid structures were obtained through a solvent-free procedure involving two consecutive thiol-ene reactions, a first one implying heptakis[6-(*tert*-butyldimethylsilyl)-2-*O*-allyl]-β-cyclodextrin and thiol-functionalized HNTs, and a second one implying the resulting βCD-HNT adduct and 1-thioglycosides. Examples on record comprise HNTs appended with mannosyl, galactosyl and lactosyl/βCD glycoclusters. These hybrids combined the inclusion properties of both HNTs and βCD and the lectin recognition abilities of the glycoligands. As a proof of concept, HNTs decorated with mannosylated-βCD functional units were found to incorporate the natural anticancer drugs silibinin (Sil) and curcumin (Cur) in the HNT and the βCD cavities respectively, while displaying high affinity towards mannose-specific lectins. The authors further confirmed the synergistic effect deriving of the co-administration of curcumin and silibinin in the 8505c thyroid cancer cell line [197] (Figure 23). The suitability of the strategy based on the attachment of CD-centered glycoclusters onto nanoparticulated platforms to improve their biocompatibility and convey lectin recognition abilities was also confirmed by Gref, Vargas-Berenguel and coworkers for the particular case of metal organic frameworks (MOFs) [198].

## 10. Conclusions and Outlook

The controlled amalgamation of cyclodextrin modules and glycan moieties provides a direct and effective way to create multifunctional glyconanomaterials with tailorable characteristics. The rich chemistry of CDs further allows programing their self-assembly and co-assembly properties with (bio)macromolecular partners to afford nanosystems that can be either pre- or post-conjugated with one or more types of glycoligands to hierarchically build the desired glyconanostructures. Exploiting host–guest strategies, enabled by multivalent displays of CD cavities in combination with high-affinity cavity-fitting motifs in the glycan partners, allows reaching high sophistication degrees with relatively low synthetic efforts. Alternatively, glycoCDs can be used as building blocks for the generation of supramolecular glyconanomaterials or to covalently modify appropriately functionalized nanoparticles. Whatever the strategy, the glycoligands become multivalently exposed at the surface of the final construct at densities that can be regulated, thus offering unlimited opportunities to tune their selectivity and affinity towards carbohydrate-binding receptors (lectins). Current applications focus on site-specific drug/gene delivery and protein separation and analysis. Considering the avant-garde developments in CD chemistry, glycobiology and nanotechnology, a much broader range of progress is expected in the near future from their synergistic blend, including fields as diverse as catalysis [199,200], bioremediation [201], sensing [202,203,204,205], diagnostics [206,207], theranostics [208,209], vaccines [210,211] or personalized medicine [212,213].
